# Improved Dye-Filling Protocol for Multiple Nematode Species

**DOI:** 10.17912/micropub.biology.001963

**Published:** 2026-01-14

**Authors:** Luke T. Geiger, Joke Evenblij, Curtis M. Loer, Yasmin H. Ramadan, Oliver Hobert

**Affiliations:** 1 Department of Biological Sciences, Columbia University; 2 University of San Diego, San Diego, California, United States; 3 Howard Hughes Medical Institute

## Abstract

Dye-filling has been used extensively to study the development and morphology of sensory neurons in the nematode *C. elegans* . Here, we report a modified dye-filling protocol that improves the robustness of amphid, phasmid, and inner labial neuron dye-filling in *C. elegans* , as well as in the nematode satellite model *P. pacificus.* Our method also allows for the consistent visualization of male specific ray sensory neurons and associated support cells in these species.

**Figure 1. Dye-filling of amphid, phasmid, inner labial, and ray neurons across multiple nematode species:**
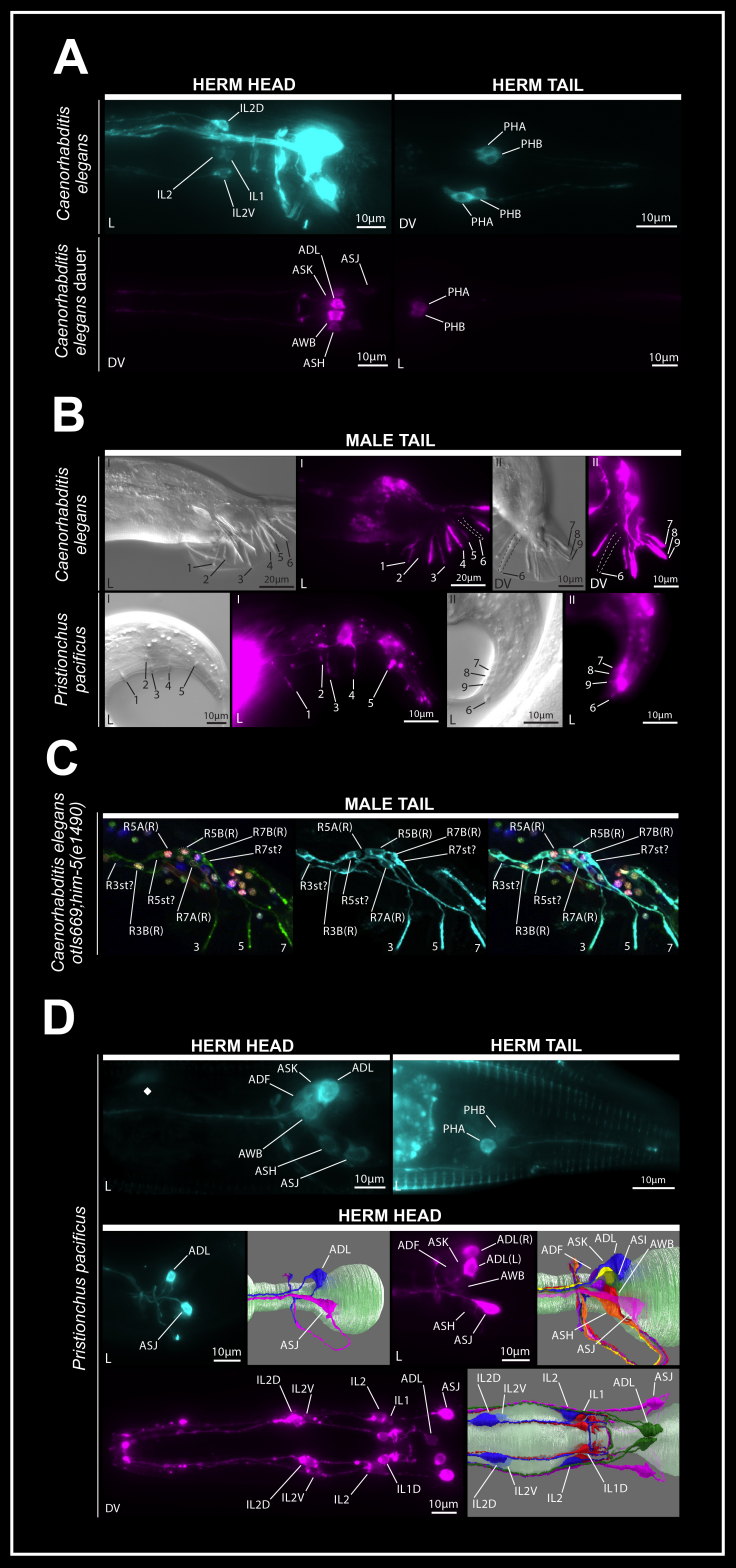
For all images, DiD: Magenta; DiO: Cyan (pseudocolor). **(A)** Representative maximum projection images of the anterior and posterior of *C. elegans* hermaphrodites (top) and SDS-selected dauers (bottom) following our modified dye-filling protocol *.* AWB is dim and occluded by ASK in the representative maximum projection image of a *C. elegans* DiD-filled dauer. **(B)** Representative maximum projection Nomarski contrast and DiD fluorescent images from the tail of young male *C. elegans* (top) and *P. pacificus* (bottom). Ray numbering for *C. elegans* and *P. pacificus* follows the conventions in **Sommer et al., 1996** and **Potts, 1910** . While some rays from the right side of the animal are visible for *C. elegans* animal I *,* only rays originating from the left side of the animal are annotated for clarity. For both image sets, Roman numerals indicate Nomarski and fluorescent image pairs originating from the same animal. **(C)** Maximum projection of dye-filled ray neurons and support structures in the posterior lumbar ganglion of a young *otIs669; him-5 ( e1490 )* *C. elegans* male (NeuroPAL). From left to right: NeuroPAL signal with slight CyOFP bleed-through in green, DiO pseudocolor signal, and merge. **(D)** Maximum projections of well-filled wild type J4/young adult *P. pacificus* amphid and phasmid neurons (top). Maximum projections of well-filled inner labial and amphid neurons in the anterior nervous system of J4/young adult *P. pacificus* animals alongside orientation-matched volumetric EM 3D renderings of visualized neurons with data from **Cook et al., 2025** (middle, bottom). Pharynx in light green. ADL and ASJ most consistently fill with dye in *P. pacificus* using our protocol, and an image representing this scenario is presented alongside an image with all amphid neurons filled for clarity. ASH, ADF and ASK are filled in many, but not all animals, using our protocol and present more dimly than ADL or ASJ when they are identified. AWB is often particularly dim and partially occluded by ADF and ASK. Neuron identity in A, B, and D was determined through relative position and projection morphology (middle). For all images, L and DV indicate lateral and dorsoventral views, respectively. All animals have wild-type genotypes unless otherwise indicated.

## Description

Dye-filling is a simple, fast and inexpensive method to visualize subsets of ciliated sensory structures in nematodes. In *C. elegans* , six pairs of amphid sensory neurons (ASK, ADL, ASI, AWB, ASH, and ASJ) and two pairs of phasmid neurons (PHA and PHB) routinely take up lipophilic dyes such as DiI, DiO, or DiD ( **Hedgecock et al., 1985; Andrews et al., 2025; Tong et al., 2010** ). By integrating into the plasma membrane of environmentally-exposed sensory cilia, these dyes provide a visualization of cellular position and morphology. This precision has allowed researchers to conduct forward genetic screens that identify loci implicated in the development and function of sensory neurons (e.g. **Neal et al., 2016; Starich et al., 1995** ). Reverse genetic experiments that assess the role of specific genes in sensory system development and function also frequently utilize dye-filling experiments (e.g. **Blacque et al., 2004; Wicks et al., 2000** ). As the failure of any given worm to dye fill during an experiment for technical reasons confounds the biological interpretability of that experiment, it is important that nematode researchers seek to continue to improve the robustness of this procedure.

Our lab seeks to develop and optimize protocols for visualizing neuroanatomy of satellite-model nematode species. In the course of attempting to dye fill nematodes that are divergent from the classic *Caenorhabditis elegans* model, we noted that modifications to the standard dye-filling protocol described in this publication not only permitted the dye-filling of those much less studied nematodes, including *Plectus sambesii* , but also improved the extent of dye-filling in the classic *C. elegans* model and the now widely-used satellite model *Pristionchus pacificus .* We describe here the usage of this novel protocol for dye-filling of *C. elegans* and *P. pacificus* and will report our dye-filling efforts of more basal nematode species in future publications.

Previous work has demonstrated that lowering the salt concentration of the solution used for dye-filling, including a detergent in M9 preparations, adding wash steps, and mechanical agitation positively affects the prevalence of dye-filling of the inner labial IL2 neurons, in addition to the amphid and phasmid neurons, in *C. elegans* ( **Tong et al., 2010** ). While most protocols for *C. elegans* call for a 1:200 dilution of dye in M9 staining solution, low-salt preparations with 10 μg/ml of stock dye solution (1:100 dilution) also facilitate the dye-filling of most *C. elegans* amphid and phasmid neurons in the dauer stage, in addition to variable filling of amphid, phasmid, IL2, and putative IL1 neurons in a number of other nematode species ( **Han et al., 2016).** Recently, a low-salt solution of 5% M9 and 1:500 DiO dilution successfully stained both amphid neurons and a variable number of body wall neurons in *Mononchus aquaticus* ( **Han et al., 2025** ). Increasing the duration of incubation during dye-filling beyond two hours – which may generally allow increased dye integration into cell membranes or induce physiological changes in nematodes relevant to dye uptake by sensory cilia - has also been demonstrated to improve the robustness of this procedure in *Steinernema hermaphroditum* ( **Garg et al., 2022** ).

Based on these prior observations, we developed a protocol that combines a low-salt incubation solution, detergent exposure, multiple pre-filling wash steps, and mechanical agitation during incubation. This modified protocol permitted the robust visualization of six pairs of amphids and two pairs of phasmids in *C. elegans* ( **Figure 1A** ). Our protocol robustly stains IL2 and IL1 neurons without requiring the addition of calcium acetate. In dauer stage *C. elegans* , our new protocol recapitulates previously reported dye-filling of five pairs of amphids except ASI, which is remodeled during this stage ( **Peckol et al., 2001, Han et al., 2016** )( **Figure 1A** ).

Although a low salt solution alone has been demonstrated to facilitate IL2 neuron dye filling in *C. elegans* , the addition of mechanical agitation during filling can also improve filling **(Tong et al., 2010)** . We report that if our low-salt protocol is performed without Triton X-100, the addition of intermittent mechanical agitation dramatically increases the proportion dye filling IL1 and IL2 neurons **(Extended Data Figure 1A )** . While we rely on manual effort by scientists to mechanically shake 1.5mL Eppendorf tubes along their long axis with moderate (see *Methods* ) force during incubation, the substitution of this device for an orbital shaker with a more well-defined cycle speed and force as outlined in ( **Tong et al., 2010** ) may further improve the consistency of dye filling results using our protocol.

Strikingly, we found that the new protocol now also fills the environmentally exposed ray sensory neurons of young male *C. elegans* ( **Figure 1B )** . While the male rays of several *C. elegans* mutant strains have been described to occasionally dye fill using FITC and M9 buffer ( **Perkins et al., 1986** ), our method allows the robust and consistent visualization of ray neurons in wild type males. We identified the type of dye filling ray neurons using the NeuroPAL transgene *otIs669* ( **Yemini et al., 2021** ), revealing mostly RnB neurons, as well as putative Rnst support cells ( **Figure 1C** ). Among the B-type ray sensory neurons, we observe all but R6BL/R to fill with dye *,* supporting previous electron micrograph assessments of these structures which showed that R6B neurons are not exposed to the environment (n = 16 animals quantified across experiments in Fig. 1B and C, where at least one other ray took up dye)( **Sulston et al., 1980** ). Filling of RnA neurons was also observed, but more intermittently **( Figure 1C )** .

While we cannot rule out that the dye filling of male rays is the result of chemical or mechanical damage, we observe that wild type *C. elegans* males monitored post-filling will often scan the length of hermaphrodites with their rays and successfully locate vulvae. Additionally, extremely sporadic *C. elegans* male ray filling was observed even after the removal of .05% Triton X-100 (but not mechanical agitation) from our protocol, suggesting that our method better facilitates – but not unnaturally so – the visualization of these structures.

We applied our protocol to dye filling defective *dyf-7 ( m537 )* mutants in which sensory dendrites of amphid and phasmid neurons fail to develop properly ( **Heiman and Shaham, 2009** ). No dye-filling of any structure was observed in either hermaphrodites or males of this genotype **(Extended Data** **Figure 1B )** , indicating that, as expected, our protocol relies on the exposure of sensory cilia to the environment.

Our modified protocol is also relevant to dye-filling of the satellite nematode *P. pacificus.* Prior experiments using an M9-based, 1:150 dilution dye-filling protocol have shown that in *P. pacificus* , ADF, ASK, ADL, AWB, ASH, and ASJ – but not ASI - fill with DiI, and that phasmid neurons were only observed to fill in dauer animals ( **Hong et al., 2019** ). Moreover, specificity of dye uptake was observed, such that DiO did not appear to fill ADF, ASK, AWB, or ASH in *P. pacificus* , while inner labial sensory neurons were not visualized with either dye in these experiments ( **Hong et al., 2019** ). &nbsp;Our modified protocol demonstrates that all three dyes (DiO, DiI, DiD) fill the same set of six *P. pacificus* amphid neurons, including ADF instead of ASI, as well as the IL1 and IL2 neurons and the phasmid neurons ( **Figure 1D ; Extended Figure 1C** ). Moreover, we also find that all male ray sensory structures in *P. pacificus* fill with dye ( **Figure 1B** ). The choice of dye did not qualitatively affect ray staining in *P. pacificus* males, and almost all males exhibited at least one ray filling across any given experimental replicate **(Extended Data Figure 1C )** . Cephalic neurons, which have not previously been described to dye-fill in wild type animals, were also observed to dye fill in some *P. pacificus* males ( **Bae et al., 2008; Perkins et al., 1986** ). The morphology and position of these cells *,* as well as the male-specificity of dye-filling indicates that these neurons are the *P. pacificus* CEM neurons.

Finally, we noticed that the number of *C. elegans* animals with filled ASI neurons decreased with shaking after quantifying the effect of multiple protocol adjustments to dye-filling **(Extended Data Figure 1A )** . As our dye-filling of *P. pacificus* included shaking but did not successfully visualize a compelling ASI neuron candidate, we asked if performing our protocol without shaking would affect ASI filling in *P. pacificus* . While we observed no new dye-filling following this modification, putative CEM neurons in *P. pacificus* males filled more consistently and robustly **(Extended Data Figure 1D )** . Further modifications to the osmolarity of the dye-filling solution, the concentration of dye used during incubation, or the intensity of mechanical agitation during incubation may further optimize the robustness of this protocol in these and other nematodes.

## Methods

*Nematode Culturing*

*C. elegans* and *P. pacificus* were reared on NGM seeded with OP50 as previously described ( **Sommer et al., 1996** ). Dauer *C. elegans* were produced by washing starved crowded plates of wild type worms with 1% SDS solution, then incubating for 15 minutes and proceeding with the dye-filling protocol at the first centrifugation step. Male *C. elegans* and *P. pacificus* were passaged as L4/J4 animals and allowed to mature overnight into young males for use in experiments.

&nbsp;

*Dye-Filling Protocol*

1.) Prepare a dye-filling incubation solution of .05% Triton X-100 and 5% M9 by volume in dd (double distilled) H20, about 8mL per sample. Prepare the solution fresh on the day of the experiment.

2.) Gather plates with animals for dye-filling. Worms should be well-fed. Rinse plates with 1.5mL of incubation solution, being careful to dispense the solution away from any lawn remaining on plates to minimize bacteria in the incubation solution.

3.) Gently agitate plates in a circular motion on the benchtop to release worms into incubation solution. Wash each subsequent plate for individual samples with the same 1.5mL incubation solution dispensed onto the first plate, passaging the solution to additional plates with a Pasteur pipette and performing the same gentle agitation.

4.) Transfer the final volume of incubation solution washed over sample plates into 1.5mL tubes. Spin down tubes at 1000g for 1 minute.

5.) Remove as much supernatant as possible, then replace up to 1mL with incubation solution. Repeat steps 4 and 5 an additional four times, for a total of five washes. Worms should be entirely free of visible bacteria or other debris; add washes as necessary.

6.) Spin down worms a final time at 1000g for 1 minute, then use a Pasteur pipette to transfer the bottom fraction of each 1.5mL tube into fresh tubes for staining. Add incubation solution up to 500μL in each staining tube.

7.) Add 10μL of lipophilic dye stock for a final concentration of about 1:50 in 500μL incubation solution.

8.) Cover tubes in foil, then shake with mild to moderate force (i.e., can barely feel and hear incubation solution hit both ends of tube) lengthwise for 2 minutes.

9.) Place tubes on a nutator at moderate speed for six hours at room temperature (~23°C), repeating step 8 every 30 minutes.

10.) Following incubation, spin tubes down at 1000g for 1 minute, then remove worms from the bottom of each tube with a Pasteur pipette. Allow worms to shed excess dye on unseeded plates for 30 minutes prior to mounting and observation. Optionally, to reduce signal from the gut, transfer worms to seeded plates for at least 30 minutes.

## Reagents

Cell Labeling Solutions:

Vybrant™ DiD Cell-Labeling Solution, Thermo Fisher Scientific, Catalog no. V22887

Vybrant™ DiO Cell-Labeling Solution, Thermo Fisher Scientific, Catalog no. V22886

Vybrant™ DiI Cell-Labeling Solution, Thermo Fisher Scientific, Catalog no. V22885

&nbsp;

**Strains**

*C. elegans* N2 wild type

*C. elegans* OH15363 : *otIs669; him-5 ( e1490 )*

*C. elegans* SP1735 : *dyf-7 ( m537 )*

*P. pacificus* PS312 wild type

## Data Availability

Description: Extended Data Figure and Legend. Resource Type: Image. DOI: https://doi.org/10.22002/fz2tg-fwz93

## References

[R1] Andrews NM, Gerten L, Edwards A, Brennan M, Reynolds Q, Bernstein S, Springer T, LaBonty M (2025). DiI Dye-Filling as a Simple and Inexpensive Tool to Visualize Ciliated Sensory Neurons in C. elegans.. J Vis Exp.

[R2] Bae YK, Barr MM (2008). Sensory roles of neuronal cilia: cilia development, morphogenesis, and function in C. elegans.. Front Biosci.

[R3] Blacque OE, Reardon MJ, Li C, McCarthy J, Mahjoub MR, Ansley SJ, Badano JL, Mah AK, Beales PL, Davidson WS, Johnsen RC, Audeh M, Plasterk RH, Baillie DL, Katsanis N, Quarmby LM, Wicks SR, Leroux MR (2004). Loss of C. elegans BBS-7 and BBS-8 protein function results in cilia defects and compromised intraflagellar transport.. Genes Dev.

[R4] Cook SJ, Kalinski CA, Loer CM, Memar N, Majeed M, Stephen SR, Bumbarger DJ, Riebesell M, Conradt B, Schnabel R, Sommer RJ, Hobert O (2025). Comparative connectomics of two distantly related nematode species reveals patterns of nervous system evolution.. Science.

[R5] Garg P, Tan CH, Sternberg PW (2022). DiI staining of sensory neurons in the entomopathogenic nematode Steinernema hermaphroditum.. MicroPubl Biol.

[R6] Han Jaeyeong, Ficca Alyson, Lanzatella Marissa, Leang Kanika, Barnum Matthew, Boudreaux Jonathan C. T., Schroeder Nathan E. (2025). Analysis of Nematode Ventral Nerve Cords Suggests Multiple Instances of Evolutionary Addition and Loss of Neurons.

[R7] Han Z, Boas S, Schroeder NE (2016). Unexpected Variation in Neuroanatomy among Diverse Nematode Species.. Front Neuroanat.

[R8] Hedgecock EM, Culotti JG, Thomson JN, Perkins LA (1985). Axonal guidance mutants of Caenorhabditis elegans identified by filling sensory neurons with fluorescein dyes.. Dev Biol.

[R9] Heiman MG, Shaham S (2009). DEX-1 and DYF-7 establish sensory dendrite length by anchoring dendritic tips during cell migration.. Cell.

[R10] Hong RL, Riebesell M, Bumbarger DJ, Cook SJ, Carstensen HR, Sarpolaki T, Cochella L, Castrejon J, Moreno E, Sieriebriennikov B, Hobert O, Sommer RJ (2019). Evolution of neuronal anatomy and circuitry in two highly divergent nematode species.. Elife.

[R11] Neal SJ, Park J, DiTirro D, Yoon J, Shibuya M, Choi W, Schroeder FC, Butcher RA, Kim K, Sengupta P (2016). A Forward Genetic Screen for Molecules Involved in Pheromone-Induced Dauer Formation in Caenorhabditis elegans.. G3 (Bethesda).

[R12] Peckol EL, Troemel ER, Bargmann CI (2001). Sensory experience and sensory activity regulate chemosensory receptor gene expression in Caenorhabditis elegans.. Proc Natl Acad Sci U S A.

[R13] Perkins LA, Hedgecock EM, Thomson JN, Culotti JG (1986). Mutant sensory cilia in the nematode Caenorhabditis elegans.. Dev Biol.

[R14] Potts F. A. (1910). Notes on the Free-Living Nematodes. Journal of Cell Science.

[R15] Starich TA, Herman RK, Kari CK, Yeh WH, Schackwitz WS, Schuyler MW, Collet J, Thomas JH, Riddle DL (1995). Mutations affecting the chemosensory neurons of Caenorhabditis elegans.. Genetics.

[R16] Sulston JE, Albertson DG, Thomson JN (1980). The Caenorhabditis elegans male: postembryonic development of nongonadal structures.. Dev Biol.

[R17] Tong YG, Bürglin TR (2010). Conditions for dye-filling of sensory neurons in Caenorhabditis elegans.. J Neurosci Methods.

[R18] Wicks SR, de Vries CJ, van Luenen HG, Plasterk RH (2000). CHE-3, a cytosolic dynein heavy chain, is required for sensory cilia structure and function in Caenorhabditis elegans.. Dev Biol.

[R19] Yemini E, Lin A, Nejatbakhsh A, Varol E, Sun R, Mena GE, Samuel ADT, Paninski L, Venkatachalam V, Hobert O (2020). NeuroPAL: A Multicolor Atlas for Whole-Brain Neuronal Identification in C.&nbsp;elegans.. Cell.

[R20] Sommer, R.J., et al. “Morphological, Genetic and Molecular Description of Pristionchus Pacificus n. Sp. (Nematoda: Neodiplogastridae).” *Fundamental and Applied Nematology* , vol. 19, pp. 511–21

